# Direct Vascular Effects of Angiotensin II (A Systematic Short Review)

**DOI:** 10.3390/ijms26010113

**Published:** 2024-12-26

**Authors:** György L. Nádasy, András Balla, Gabriella Dörnyei, László Hunyady, Mária Szekeres

**Affiliations:** 1 Department of Physiology, Faculty of Medicine, Semmelweis University, 37-47 Tűzoltó Street, 1094 Budapest, Hungary; nadasy.gyorgy.laszlo@semmelweis.hu (G.L.N.); balla.andras@semmelweis.hu (A.B.); hunyady.laszlo@semmelweis.hu (L.H.); 2 HUN-REN-SU Molecular Physiology Research Group, Hungarian Research Network, Semmelweis University, 1094 Budapest, Hungary; 3 Department of Morphology and Physiology, Faculty of Health Sciences, Semmelweis University, 17 Vas Street, 1088 Budapest, Hungary; dornyei.gabriella@semmelweis.hu; 4 Institute of Molecular Life Sciences, HUN-REN Research Centre for Natural Sciences, 2 Magyar Tudósok Körútja, 1117 Budapest, Hungary

**Keywords:** angiotensin II, renin-angiotensin system, angiotensin receptors, vascular effects, vascular remodeling

## Abstract

The octapeptide angiotensin II (Ang II) is a circulating hormone as well as a locally formed agonist synthesized by the angiotensin-converting enzyme (ACE) of endothelial cells. It forms a powerful mechanism to control the amount and pressure of body fluids. All main effects are directed to save body salt and water and ensure blood pressure under basic conditions and in emergencies. All blood vessels respond to stimulation by Ang II; the immediate response is smooth muscle contraction, increasing vascular resistance, and elevating blood pressure. Such effects are conveyed by type 1 angiotensin receptors (AT_1_Rs) located in the plasma membrane of both endothelial and vascular smooth muscle cells. AT_1_Rs are heterotrimeric G protein-coupled receptors (GPCRs), but their signal pathways are much more complicated than other GPCRs. In addition to G_q/11_, the G_12/13_, JAK/STAT, Jnk, MAPK, and ERK 1/2, and arrestin-dependent and -independent pathways are activated because of the promiscuous attachment of different signal proteins to the intracellular G protein binding site and to the intracellular C terminal loop. Substantial changes in protein expression follow, including the intracellular inflammation signal protein NF-κB, endothelial contact proteins, cytokines, matrix metalloproteinases (MMPs), and type I protocollagen, eliciting the inflammatory transformation of endothelial and vascular smooth muscle cells and fibrosis. Ang II is an important contributor to vascular pathologies in hypertensive, atherosclerotic, and aneurysmal vascular wall remodeling. Such direct vascular effects are reviewed. In addition to reducing blood pressure, AT_1_R antagonists and ACE inhibitors have a beneficial effect on the vascular wall by inhibiting pathological wall remodeling.

## 1. Introduction

Angiotensin II (Ang II) is an octapeptide, the main function of which is to keep and elevate blood pressure, both in basic conditions and in emergency situations. This is performed by saving body salt together with body water, as well as by constricting blood vessels. In addition to its main effects, Ang II has proliferative, proinflammatory, proapoptotic, and profibrotic effects on the vascular wall and other tissues and organs. With its diverse actions, Ang II is an important pathogenetic factor in several pathological states [1]. The inhibition of its production or action is performed by drugs used regularly by a substantial part of the population in developed countries [2].

In the present review, we intend to give a short but, as much as it is possible, full and concise picture of the direct effects of Ang II on blood vessels, a clinically and scientifically very important topic, to provide a full view of the main processes, from elementary molecular reactions to the complex pathological events. Meanwhile, because of limited space, we had to reduce many interesting and potentially important details. Interested readers are advised to read excellent papers, among other review papers, on separate areas of molecular, cellular, pharmacological, and pathological studies.

## 2. Renin

Renin is a highly selective aspartic protease enzyme. It starts the formation of Ang II, cleaving the decapeptide angiotensin I (Ang I) from the plasma protein angiotensinogen. Ang I, in turn, will be subjected to the action of angiotensin-converting enzyme (ACE), which produces the active octapeptide. In the extrauterine life, renin is mostly expressed and released from the granular cells of the juxtaglomerular apparatus (JGA) of the kidney [3,4]. In emergency states, mesangial cells and smooth muscle cells of the afferent arteriole contribute to renin production [5]. While renin-expressing cells can be found in different parts of the body [6], even the vascular wall expresses it [7], most Ang II will be formed by local ACE from local Ang I formed by circulating renin [3,4]. In addition to its enzymatic function, renin and prorenin bind to their specific prorenin single-transmembrane receptor (P)RR. Such receptors are expressed on the surface of vascular smooth muscle cells and induce proliferation through the extracellular signal-regulated kinases ERK1, ERK2, and Akt pathways [8,9,10]. A pathomechanistic role in the development of atherosclerotic plaques has been proposed based on studies on cultured human vascular smooth muscle cells [11].

## 3. Angiotensinogen

Angiotensinogen is a plasma protein synthesized by the liver and is the substrate of renin. Angiotensinogen mRNA was found in the periadventitial brown fat tissue of the rat aorta and in the adventitia but not in the media [12]. Several minor inherited variations of the angiotensinogen protein molecule could be identified despite the fact that the molecule itself is phylogenetically well-conserved. They affect the incidence of cardiovascular diseases [13]. The angiotensinogen molecule per se (with and without its section cleaved off by renin) has inherent biological activity. An antiangiogenetic effect has been proven on cultured endothelial cells [14]. Increased expression inhibited the growth of the wall of renal arteries even without a concomitant rise in blood pressure or elevation in Ang II levels [15]. Hypo-angiotensinogenic low-density lipoprotein receptor (LDLR)-knockout (LDLR^−/−^) mice, fed on a Western-type diet, had reduced blood pressure, cholesterol levels, and less extensive atherosclerosis compared with their counterparts with normal angiotensinogen expression [16,17]. No molecular receptor for angiotensinogen has yet been identified.

## 4. Angiotensin Converting Enzyme (ACE)

ACE is a zinc metalloproteinase; by its action, it is a partially specific dipeptidyl peptidase. It converts the product of renin cleavage, angiotensin I, into the active octapeptide, angiotensin II. ACE is expressed in many cell types, tissues, and organs [1,18]. It is abundantly expressed in the plasma membrane of endothelial cells [19], producing most of the Ang II with vascular effects [1]. ACE is expressed in vascular smooth muscle cells in small amounts under basal conditions, but expression can be stimulated [20]. The expression of ACE is substantially elevated in human carotid atherosclerotic plaques [21]. Smooth muscle ACE plays an important role in the pathomechanism of arteriosclerosis [22]. Plasma ACE activity poorly reflects the rate of Ang II production, as most ACE molecules are bound to the plasma membranes of endothelial cells by their hydrophobic C terminal transmembrane regions. The distribution of ACE in the whole body can be examined by micro-SPECT using the radioactive tracer ^99m^Tc-Lisinopril in experimental animals [23]. Some genetic polymorphisms of the ACE enzyme are associated with ventricular fibrosis and heart failure [24].

An interesting connection between metabolic state and microcirculatory function with substantial pathologic consequences is that in obese rats kept on a high-fat diet, ACE activity was elevated in the coronary arterioles. This effect elicited a reduced endothelium-dependent vasorelaxation in response to bradykinin [25].

The control of the expression of the elements of the renin–angiotensin system (RAS) in the blood vessel wall is still not well known. In the promoter region of ACE, a mechanosensitive element is present, and ACE expression is downregulated by normal arterial levels of flow-shear stress [26,27]. Activation of AMP-activated protein kinase (AMPK), p53 phosphorylation, and elevation in the levels of a microRNA (miR-143/145) are involved in the control of ACE expression, interfering with its posttranscriptional processing [28].

An endothelial cell population with a lower level of expression of CD 143 (ACE) turned out to be a more active contributor to angiogenetic processes (endothelial sprouting) than other smooth muscle cells. Sprouting is an important step in the restoration of tissue blood flow following ischemia [29].

## 5. Angiotensin II (Ang II)

Ang II is an octapeptide; it is the main agonist fulfilling the basic functions of the RAS. Ang II is known as one of the most powerful vasoconstrictor agents. Maximum effects are in the range of those for the alpha-adrenergic agonist phenylephrine, the thromboxane (TX) A_2_ agonist U46619, or endothelin. Threshold concentrations are around 1 nM, and EC50 concentrations are around 100 nM. These concentrations are much higher than in vivo Ang II concentrations in the blood plasma of healthy subjects, which are around 20 pM/lit (as measured by mass spectroscopy) [30]. However, we should not forget that because of the endothelial localization of ACE, local concentrations in and around the vessel wall can be much higher than that. Receptors for Ang II are present in all vascular walls studied, in endothelial and vascular smooth muscle cells alike, ensuring a direct effect on the vasculature. In addition, through its extravascular effects, blood vessels will also be indirectly affected. AT_1_R is the main receptor mediating the effects of Ang II in blood vessels and elsewhere. Other agonist peptides of the RAS system also have direct vascular effects. We have to remark here that ACE2, a carboxypeptidase, degrades Ang II to form Ang (1–7), which stimulates AT_2_R and Mas receptors. This will result in opposite vascular effects: vasodilation, decreased fibrosis, and reduced inflammation in atherosclerotic plaques counteracting the actions of Ang II [31,32]. Further studies are needed to reveal the balance of these actions in different cell types and under different conditions.

## 6. Angiotensin Receptors

**Figure 1:**
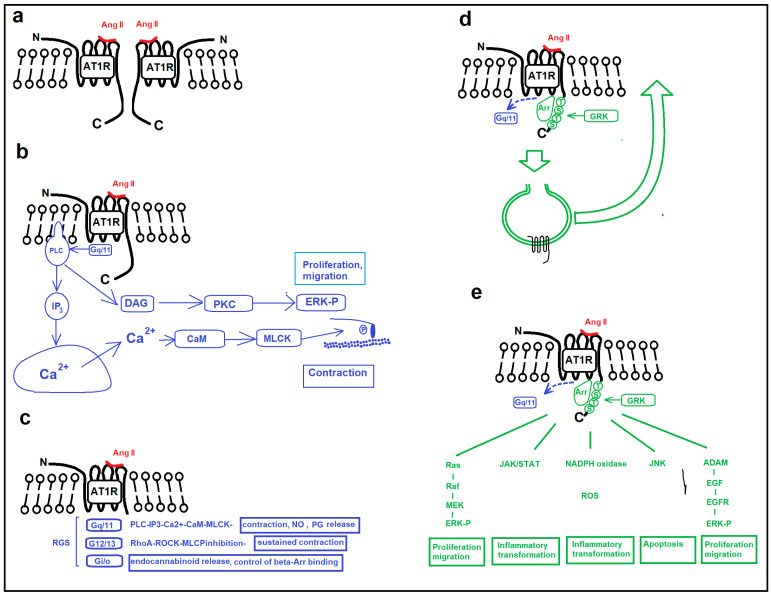
Signal pathways of the angiotensin type 1 receptor (AT_1_R) in vascular cells. (**a**). Dimerization of the receptor molecule might cause mutual inhibition. (**b**). The “classical” heterotrimeric G protein activation pathway (with G_αq/11_) induces smooth muscle contraction. Increased intracellular Ca^2+^ and membrane DAG activate PKC enzyme subtypes with diverse effects. (**c**). “Promiscuous” association of the intracellular G protein binding site with other heterotrimeric G proteins, altered specificity is supposed to be controlled by different RGS proteins. (**d**). Receptor internalization, digestion, or recycling to the membrane after β-arrestin binding. threonin (T) and serine (S) phosphorylations by GRK kinases at the intracellular C terminal loop promote β-arrestin binding. The binding of heterotrimeric G proteins to their binding site will be prevented. (**e**). Diverse intracellular signal pathways are activated by C terminal phosphorylations and β-arresting binding. Abbreviations: Ang II, angiotensin II; AT_1_R, angiotensin type 1 receptor molecule; N, N terminal; C, C terminal; PLC, phospholipase C; IP_3_, inositol triphosphate; CaM, calmodulin; MLCK, myosin light chain kinase; G_q/11_, G_12/13_, G_i/o_, heterotrimeric G proteins with corresponding α subunits; RGS, regulator of G protein signaling; NO, nitrogen oxide; PG, prostaglandins; RhoA, small GTPase; ROCK, Rho-associated protein kinase; Arr, β-Arrestin; GRK, G protein-coupled receptor kinase; Ras, Raf, MEK, components in the MAP (mitogen-activated protein) kinase cascade; ERK-P, extracellular signal-regulated kinase, phosphorylated (activated) form; JAK/STAT, cytokine signal pathway; NADPH oxidase, main source of reactive oxygen species (ROS) in mammalian tissues; JNK, apoptotic signal protein; ADAM, membrane metalloproteinase, it cleaves off membrane-bound EGFR ligand; EGF, released peptide with EGF (epithelial growth factor) activity; EGFR, epithelial growth factor receptor. Blue color marks signal pathways through the G protein binding site, green color marks signal pathways through the C terminal loop.

Angiotensin receptor type 1 (AT_1_R) mediates most actions of Ang II. It is a 7-transmembrane (7TR) heterotrimeric G protein-coupled receptor (GPCR); for the steric structure, see refs. [33,34]. Unusually for such receptors it can promiscuously attach to different heterotrimeric G proteins with its intracellular domain. G_q/11_, G_12/13_, and G_i/0_ proteins can be bound and activated. It seems to be promising that a series of control proteins have been identified whose subtypes determine which G protein will be bound to the G protein binding intracellular loops of the receptor molecule (Figure 1c) [35,36]. The “classical” G_q/11_/phospholipase C/inositol triphosphate (IP_3_)/Ca^2+^ pathway (Figure 1b) elicits immediate vascular smooth muscle contraction as well as endothelial nitric oxide (NO) production. In addition, elevated Ca^2+^ levels activate protein kinase C (PKC); kinase activity affects a series of proteins, and the result is epidermal growth factor (EGFR)-independent ERK activation with massive alterations in the protein expression. Other enzymes and signal proteins activated are phospholipase D (PLD), phospholipase A2 (PLA2), adenylate cyclase, PI3K/Akt, ERK1/2- mitogen-activated protein kinases (MAPK), Janus-kinase signal transducer (JAK-STAT), and nicotinamide adenine dinucleotide-phosphate (NADPH) pathways. Activation is mostly through the intracellular C terminal of the receptor molecule and β-arrestin binding. These result in substantial inflammatory, proliferative, apoptotic, and fibrotic effects [33,37,38,39,40,41,42,43,44,45,46,47,48]. The complexity of the intracellular pathways raises hopes that drugs selectively inhibiting the different functions of Ang II can be developed: biased” agonists and antagonists [49]. AT_1_R is a typical receptor with tachyphylactic properties; repeated stimulation induces less effective action. This can be explained by the long residence time of Ang II on the receptor molecules, which determines sustained action and prolonged receptor internalization and, thus, reduced receptor recycling [50].

Receptor function is affected by frequent homo- and heterodimerization (Figure 1a) of the membrane-bound AT_1_R molecules [47,51]. Revealing the pathological significance of such dimer formations in vascular cells is a promising area; however, further studies are needed.

The identification of a membrane protein specifically attaching to the carboxyterminal third intracellular loop of the AT_1_R molecule, the angiotensin receptor-associated protein (ATRAP) [52], raised hopes that diverse functions of the receptor could be specifically inhibited this way [53]. The carboxyterminal region is responsible, among others, for the activation of the JAK/STAT “cytokine” pathway, which is an important component in pathological vascular wall remodeling processes. In transgenic mice, by overexpressing ATRAP in the vascular wall, less vascular hypertrophy and superoxide damage were observed during Ang II infusion-induced hypertension while physiologically important functions were maintained [54].

Desensitization of the activated receptor is an important factor in the control of Ang II activity. After about ten minutes of attachment, the hormone receptor complex will be internalized, and only a quarter of it will be recycled to the membrane. First, the intracellular C terminal of the molecule will be Ser/Thre phosphorylated. As no autophosphorylation mechanisms exist, other kinase molecules (protein kinase C, G protein-coupled receptor kinases GRK2, GRK4, GRK5, and phosphoinositide 3-kinase; PI3K) will perform this multiple phosphorylation. As a result, the beta-arrestin molecule can attach and induce internalization but at the same time activate alternative (non-heterotrimeric G protein-dependent) signal transduction cascades [42,44,46,48,55,56,57]. The attachment of the beta-arrestin molecule to the phosphorylated intracellular C terminal loop inhibits the attachment of the heterotrimeric G protein to its intracellular binding site while it initiates endocytosis (Figure 1d). The attached beta-arrestin will activate the MAPK, JAK/STAT, and Jnk pathways (Figure 1e), important components of pathological wall remodeling. Several lasting effects of Ang II will be induced by the coactivation of the EGFR (epidermal growth factor receptor. A plasma membrane metalloproteinase (ADAM) is activated, which cleaves off a peptide with EGF activity and, in turn, stimulates the EGFR receptor. In vascular smooth muscle cells, EGFR stimulation further activates the ERK1/2, JAK/STAT3, PI3K (phosphatidyl inositol 3 kinase), and PLCγ (phospholipase C gamma) pathways, and also the light chain of the myosin molecule [58]. A substantial smooth muscle cell proliferation and migration effect through the PI3K/Akt signal pathways seems to be provided through the coactivation of the EGFR receptor [42,59].

As we mentioned above, drugs with “biased effects” could be developed with different effects on the G protein and on the C terminal/β-arrestin-dependent intracellular signal pathways. Biased antagonists of the AT_1_R effectively inhibit the activation of the G_q/11_/PLC-IP_3_ pathway, maintaining the drugs’ antihypertensive action, while the arrestin-dependent mechanisms will not be affected [51,60]. Biased effects of the Ang II analog SII have been demonstrated in adrenal cells by Lymperopoulos [61]. It activated the β-arrestin pathway but not the G protein pathway. Modification and stabilization of the receptor structure have been identified as the explanation for this biased effectivity [62]. Advanced computational structure prediction techniques revealed that altered transitional probabilities between different semi-stable steric conformations of the receptor molecules in the presence of the biased agonist could be in the background [60,63]. Ferraino [64] has demonstrated that several angiotensin receptor blockers in the clinical practice (candesartan, valsartan) inhibit more the β-arrestin pathway than the G protein pathway, at least in adrenocortical and ventricular tissue, and it is logical to call them “biased inverse agonists”. Similar effects and their potential beneficial consequences have yet to be demonstrated in vascular tissue.

Angiotensin receptors are present, and they are active everywhere in blood vessels [18,65]. This was first proven by the rather surprising effectiveness of the ACE inhibitors in controlling blood pressure in patients with essential hypertension without elevated renin or Ang II plasma levels [66].

A promising new theory of the development of hypertension and sustained vasoconstriction in pre-eclampsia has been suggested by Bian et al. [67], who found that autoantibodies against the AT_1_R could be recovered from the blood plasma of affected pregnant females and these autoantibodies interfered with the desensitization of the receptor molecule. There is no explanation, however, as to why the removal of the placenta so quickly results in a pressure drop, as it is found in clinical practice.

There are disturbingly scanty data on the amount of Ang II receptors in different tissues and under different conditions. Promising initial results are that the distribution of AT_1_Rs in the body can be visualized using radioactively labeled antagonists. Images of radioactivity have been recorded by PET/CT [23].

The sequence of the human AT_1_R genome has been determined by Guo et al. [68]. A sophisticated mechanism seems to control the expression of the human AT_1_R. Four (or more) alternatively spliced mRNAs are transcribed in human tissues. Splice variants are physiologically and pathologically regulated; transforming growth factor-beta (TGFβ)-treatment resulted in the upregulation and downregulation of different splice variants. Glucocorticoid regulatory element (GRE) sequences have been identified in the rat AT_1_R promoter region, and the presence of GRE half-elements has been observed in association with the human gene [69]. In cultured human coronary endothelial cells, oxidized low-density lipoproteins increased the expression of the AT_1_R protein, which was connected to the action of NF-κB [70].

The complexity of AT_1_R receptors is shown that they are among the few mechanosensor molecules of the vascular wall. The physiologically very important myogenic contraction (stretch response) of resistance arterioles, at least partially, depends on them. The G_q/11_/ PLC/ic. Ca^2+^ pathway is activated, followed by cytoskeletal rearrangement. Steric changes of the receptor molecule itself induced by forces acting at cellular levels have not been described yet [71,72,73].

Cellular actions through AT_1_R stimulation are not only dependent on the activation of the diverse intracellular signal transduction pathways described above. Coactivation and transactivation processes mediate further important functions. Nitric oxide, prostanoids, and endogenous cannabinoid substances are released from Ang II-activated vessels [74,75,76,77,78]. Transactivation of the epidermal growth factor receptors (EGFRs) amplifies the many biological effects of Ang II on vascular cells [47,58].

The signal pathways of the AT_1_R receptor molecule are schematically shown in Figure 1.

We must not forget that vascular wall remodeling induced by Ang II will always be a double effect: the result of a direct effect on the cellular components of the vessel wall and of the mechanical effect of sustained elevated blood pressure. Studies on isolated cells and cultured cells should be completed with studies on vascular preparations under close-to-real mechanic conditions and in vivo. Direct vascular effects are analyzed in this paper. Pathologic wall remodeling effect was observed in low-dose Ang II-infused mice with only moderate blood pressure elevation [79]. In mice, where two molecular subtypes of the receptor exist, smooth muscle AT_1a_R silenced animals had limited arterial wall damage during chronic Ang II infusion, even if blood pressure elevation was similar to that reached in the wild type [80].

There is an increasing body of evidence that Ang (1–7), the product of the ACE2 enzyme, which mostly stimulates not type 1 but type 2 angiotensin receptors (AT_2_Rs) in different parts of the cardiovascular organs (and kidney), forms a system that counteracts and reduces the effects of Ang II exerted on AT_1_Rs, resulting in vasodilation, inhibition of smooth muscle migration, and endothelial protection [81,82,83]. Ang (1–7) can also stimulate an archaic group of GPCR peptide receptors, the Mas receptors, which are also expressed in many vascular tissues, resulting in vasorelaxation, the stimulation of angiogenesis, and a reduction in oxidative stress and inflammation, moderating the detrimental vascular effects of Ang II [84]. Further studies are needed to determine the amount of these two subtypes of angiotensin receptors in different cardiovascular (and other) tissues, the means of their expression control, and their functional significance in different normal and pathological states.

## 7. Endothelial Effects

The relatively high (summed) endothelial mass of the body is a main contributor to the function of the RAS. It is the main source of the activity of ACE [85], the enzyme producing Ang II. This ensures higher Ang II concentrations in the vascular wall than blood-borne Ang II alone (“local RAS”). In addition to producing Ang II, endothelial cells, at the same time, also form a very important cellular target for this hormone. Endothelial cells, in addition to ACE, express AT_1_R [38,39,86]. Ang II activates endothelial vasodilation processes. Stimulation of endothelial AT_1_Rs by Ang II, similar to other G_q/11_-associated vasoconstrictors, induces intracellular calcium elevation in the endothelial cells, which results in the release of NO and consequent vasodilation. This moderates the direct vasoconstrictor effect of Ang II on the neighboring vascular smooth muscle cells. This moderating effect is diminished in the disturbed endothelium, which seems to be an important mechanism in several vascular pathologies [87]. A higher than optimal, lasting stimulation of endothelial cells by Ang II (and endothelin), however, can induce the uncoupling of the endothelial nitric oxide synthase (eNOS) enzyme from its cofactor tetrahydrobiopterin, reducing the amount of available NO [88]. Sustained incubation of endothelial cells with Ang II initiated protein nitration processes in them through a selective AT_1_R/NADH/NADPH oxidase pathway [89]. It is in good accord with the experimental gerontological observations performed by Li et al. [90], who found that in contrast to temporary stimulation, senescent endothelial cells treated chronically with Ang II had decreased viability, they increased expression of genes characteristic for senescence and chronic inflammation (beta-galactosidase, cytokines, and cell adhesion molecules) and their proneness for apoptosis was increased. Valsartan, the selective AT_1_R blocker, induced partial improvement of this process. These results, obtained in human umbilical endothelial cells (resembling endothelial stem cells), however, could be only partially confirmed on well-specialized adult coronary endothelial cells.

The local RAS in aged arteries induces dysfunction of the endothelial dilator system and is responsible for a substantial part of the rigidity [7]. Among the endothelial proteins that are expressed in response to Ang II is the plasminogen activator inhibitor (PAI-1), forming a direct connection between the RAS and the blood clotting systems, altering the fibrinolytic balance [91].

In addition to AT_1_R, AT_2_R is also expressed in endothelial cells. They have an opposite action regarding their effect on endothelial superoxide formation. While stimulation of the AT_1_Rs elevates superoxide production, AT_2_Rs attenuate this effect [38,92]. AT_2_R stimulation inhibits the proliferation of cultured endothelial cells and alters their extracellular connective tissue production pattern into a less growth-permissive form. Thrombospondin and fibronectin expression are increased [93]. Ang II elevates the matrix metalloproteinase 2 secretion of endothelial cells; this effect seems to also be mediated by the angiotensin type 2 receptors [94].

In 2004, Kohlstedt et al. proposed the surprising theory that in human and porcine endothelial cells, the ACE molecules themselves can serve as “outside-in” signal transduction molecules despite their short intracellular domains and activating the CK2/JNK pathway [95].

## 8. Vascular Smooth Muscle Cell and Contractile Effects

Several components of RAS are expressed in the vascular smooth muscle [65]. While endothelial cells are most abundant in ACE, vascular smooth muscle cells also express this enzyme [20]. Pulsatile pressure elevates the expression of the ACE in arterial smooth muscle cells [96]. The ACE expression is characteristically elevated in smooth muscle cells of the atherosclerotic lesion, and this is thought to form an important pathomechanistic factor [22].

The effect of Ang II stimulation on the activity of different signal pathways has been investigated in a recent paper from our laboratory in cultured rat aortic smooth muscle cells [97]. Two hours of stimulation significantly elevated the pathway activity of the vascular endothelial growth factor (VEGF), tumor necrosis factor-alpha (TNFα), TGFβ, PI3K, and NFκB, but the MAPK and EGF pathways were most effectively involved. Further analysis has shown that deactivation of the phosphorylated MAPKs is also controlled. From dual phosphatases, the control of subtypes 5, 6, 10, and 4 are upregulated by Ang II. What is very interesting is that the time course of the elevated activity of these phosphatases is subtype-specific; while DUSP5 and DUSP10 show a sharp maximum at 2 h, DUSP6 shows a lengthy 6-h long effect. Further, the old lingering question about the contribution of EGFR to the trophic effect of Ang II could be solved; a substantial protein expression-enhancing effect of Ang II remained even after silencing the EGF receptor. There seems to be a massive interaction between different agonists in their control of vascular smooth muscle cell expression processes. According to Dubourg’s [98] opinion, however, activation of AT_1_R alone does not induce substantial expression changes in the vascular smooth muscle transcriptome, but Ang II substantially modifies the expression effects of EGF and of the TXA_2_ agonist U46619. The Ang II concentrations they used (10 nM), however, might be less than the in vivo local concentrations produced by local ACE. All these effects underline the significance of making expression studies under real but strictly controlled in vivo conditions.

Angiotensin II concentrations in the blood plasma of healthy individuals are around 20 pmol/L [30]. Animal models have been criticized on the basis that enormously high amounts of Ang II are usually infused into the animals, sometimes over 400 ng/min/kg body weight [99]. The vasoconstriction elicited by Ang II is modified by substances released from the vascular wall under stimulation; NO is released from endothelial cells, and endocannabinoids and vasorelaxant prostanoids moderate, while vasoconstrictor prostanoids further elevate vasoconstriction [75,76,77,78].

AT_1_Rs are present in the smooth muscle plasma membrane of practically all vessels. The “classical” G_q/11_/PLCß/IP_3_/ic. Ca^2+^/calmodulin/myosin light chain kinase (MLCK) signal cascade activates myosin light chain phosphorylation and induces powerful contraction. However, G_12/13_-Rho/Rho-kinase (ROCK) and L-type Ca^2+^ channel-associated mechanisms are also present [39]. The parallel G_12/13_ activation inhibits the MLC phosphatase through the GTPase Rho/ROCK cascade mechanism. This ensures the sustainability of the achieved contraction, as vascular control mechanisms usually do not work in a few seconds but in several hundred seconds [100]. Receptor inactivation can be through arrestin-dependent and arrestin-independent mechanisms. The GRK receptor phosphorylation/β-arrestin binding followed by internalization is one essential pathway. There are substantial hopes that this mechanism could open new possibilities for pharmacological interventions to neutralize this important pathological component of vascular wall remodeling [41,47,100].

Recent studies have revealed that vascular smooth muscle AT_1_R receptors have an additional function in controlling vascular tone, at least in cerebral, renal, and mesenteric vessels. They work as mechanosensors, sensing circumferential wall stretch, and have a crucial role in setting the myogenic tone of resistance vessels through a G_q/11_ coupled mechanism [71,72].

The physiological significance of the continuously produced Ang II is shown by Christie’s observation, who found a substantial pressure drop in toxicological patients after ACE blocker overdosage [101]. The vessel contractile action of Ang II will be practically life-sustaining in many critically ill patients. With the exhaustion of the reserve capacities of the baroreceptor reflex, no further capacity is at hand to elevate the sympathetic tone; renin released in high amounts and Ang II will be important components of keeping blood pressure at life-sustaining levels, at least for a while [102].

A new component of the detrimental action of Ang II on vascular smooth muscle cells has been identified in our laboratory; it elevated the expression of the enzyme cholesterol-25-hydroxylase massively (by 50-fold) [103]. The product of this enzyme is a known stimulant of atherosclerotic processes in the arterial wall.

In addition to its short-term vasoconstricting effects, Ang II induces remodeling of the vascular wall. Sustained stimulation with Ang II induces vascular smooth muscle cell transformation mostly through the MAPK pathway into the secretory-migratory phase [104,105]. Chronic inflammation and apoptosis are the results, important components of vascular wall damage in hypertension, atherosclerosis, aortic aneurysms, and low extremity chronic venous diseases [39,41,106,107,108].

Ang II can be formed from Ang I in addition to ACE with the contribution of chymase enzymes. Vascular smooth muscle cells and also mastocytes present in inflammatory vascular walls express such enzymes. However, large differences between different mammalian species make it hard to determine their significance in forming Ang II in the vascular wall [109].

## 9. Hypertensive Arterial Wall Remodeling

Ang II is an important component in elevating blood pressure in patients with essential hypertension. This is convincingly demonstrated by the widely observed effectivity of RAS inhibitors as antihypertensive drugs. Such drugs are taken regularly by a substantial part of the population, especially by the elderly in developed countries [2]. The hypertensive remodeling of the arterial wall forms the most important risk for sustained, untreated patients with hypertension. Nevertheless, it is still not fully understood to what degree hypertensive wall remodeling is responsible for the direct action of Ang II on the cells of the vascular wall and to what degree it is responsible for mechanical stress [110,111]. The direct vascular damaging effect of Ang II is supported by the serious chronic inflammatory cellular mechanisms activated by it in the cellular components of the vascular wall, even under controlled in vitro conditions (without the presence of any mechanical tension). An analogy with the in vivo pathological process is obvious. Further, the clinical effectivity of RAS inhibitors is more than can be expected based on their blood pressure-reducing effect [89,112]. Simon G. et al. [113] first realized that the chronic infusion of rats with Ang II in low doses was insufficient to induce blood pressure elevation. Still, the vascular damage was present. This can be attributed only to the direct vascular damaging effect of this agonist in vivo. In our laboratory, Ang II was infused into rats in continuous hypertensive doses (150 ng/kg/min) for 4 weeks. After cessation, blood pressure partially recovered, but the reduced in vivo diameter, increased wall thickness and increased rigidity of a musculocutaneous small artery did not fully recover [114]. It is worth mentioning that, surprisingly, this new in vivo reduced lumen diameter corresponded to the new reduced in vivo local flow in the resistance artery segment. A lack of recovery of the inwardly remodeled cerebral arterioles after chronic Ang II infusion has also been found by Sabharwal et al. [115]. In a recently published work from our laboratory, after chronic Ang II infusion, a remodeling of the coronary resistance artery network geometry in rats was also observed: a number of parallel connected vessels in the 200 µm diameter range was reduced (“rarefaction”) and the number of morphologically aberrant branchings (triple branches, aberrant branching angles, undulating segments, and segments with uneven lumen diameters) was increased [116].

The continuous presence and activity of local ACE in the vascular wall in the process of pathological wall remodeling was proven by Husarek et al. [117], who, in db/db type 2 diabetic mice, could prevent inward remodeling of coronary arterioles by inhibition of the AT_1_R.

In vascular smooth muscle cells, overstimulation by Ang II, in addition to the “classical” G_q/11_ pathway, induces the activation of the G_12/13_, resulting in abnormal guanosine exchange factor (GEF), RhoA/ROCK activation and inhibition of the myosin light chain phosphatase (MLCP). Acto-myosin cross-bridges in vascular smooth muscle cells will be more stable, and there will be a sustained vasoconstriction elevating vascular resistance [118].

In a recent clinical review on ACE inhibitors, angiotensin II receptor blockers (but also calcium channel blockers) have been found to be more effective in reducing in vivo arterial stiffness than other antihypertensive drugs [119].

In the case of the very important hypertensive remodeling of the blood vessel wall, we can conclude that the direct cellular effect of Ang II and its indirect effect through the elevated blood pressure are damaging the vessel parallel; the RAS inhibitors will target both mechanisms, which explains their beneficial clinical effectivity.

## 10. Chronic Inflammation

Modern vascular pathology attributes great significance to chronic inflammation in the wall during many pathologic processes [120]. The long-term chronic inflammatory effects of Ang II are not surprising if we take into consideration the phylogenetic developmental tree of the angiotensin receptors. This suggests a common origin and substantial homology with chemokine receptors. Genes are located in the same chromosomal regions as chemokine receptor genes in mammals [121]. A further connection with inflammatory processes is that during its promiscuous signaling pathways, the JAK/STAT pathway can be activated [42,47,122]. Ang II induces the release of cytokines, among them the interleukin (IL)-6 in the vessel wall, which in turn further activates the JAK/STAT inflammatory pathway and elevates the level of the NF-κB intracellular inflammatory signal protein. This mechanism provides a self-sustaining chronic inflammatory mechanism leading toward atherosclerosis [123].

Ang II stimulates inflammatory remodeling of both endothelial and vascular smooth muscle cells. On the luminal surface of the endothelial cells, adhesive proteins are expressed (ICAM-1, VCAM-1), which stimulate white blood cell rolling, adhesion, and migration into the wall. In vascular smooth muscle cells, binding of the agonist to the receptor will be followed by the binding of arrestin to the intracellular C terminal segment. This activates the endocytic pathway and several signaling pathways, resulting in altered protein expression. Increased production of cytokines and reactive oxygen species is followed by the activation of the inflammatory cytosolic signal protein NF-κB. This latter elevates the expression of inflammatory enzymes such as cyclooxygenase (COX)2 [124]. The inflammatory transformation of vascular smooth muscle cells governed by the intracellular signaling of NF-κB also results in increased production of the enzymes degrading the existing connective tissue (matrix metalloproteinases; MMPs). The smooth muscle cells themselves produce inflammatory cytokines, among them TGFß, acting together with the invading white cells. Secretory phase smooth muscle cells cease to synthesize the contractile proteins and elastin; protocollagen will be synthesized instead. The process ends with fibrosis, cellular apoptosis, and acellularity, as well as a massive loss of vascular elasticity [125,126]. Such processes are present in atherosclerosis, where irritating cholesterol accumulation initiates local chronic inflammatory processes.

## 11. Vascular Wall Remodeling, Atherosclerosis

There seems to be an agreement that local Ang II, if not itself initiates the arteriosclerotic lesion [127], promotes its appearance both by its direct and indirect effects, and amplifies the sclerotic process [82,125]. Several publications have described the important direct pathogenetic effect of Ang II in the formation of atherosclerotic lesions [22,79,125,128,129,130,131,132,133,134].

Human atherosclerotic lesions express ACE in high amounts [21,135], demonstrating the presence of Ang II in them. Atherosclerosis is promoted by infusion of Ang I; this effect is dependent on the presence of ACE, proving the pathological role of Ang II. In atherosclerotic lesions, leukocyte ACE contributes to high ACE activity [128]. The production of local Ang II is performed in atherosclerotic lesions through the alternative chymase pathway [136]; its pathological significance has yet to be established.

In smooth muscle cells (and also in macrophages) of human coronary atherosclerotic lesions, AT_1_R was abundantly expressed, proving the potential contribution of Ang II to their formation and maintenance [137]. AT_1_R blocking agents were able to reduce the activity of the connective tissue degrading enzymes MMP-1 and MMP-8 in carotid atheromas, improving plaque stability. Unfortunately, enzymes degrading elastin were not involved in this beneficial pharmacological process [138]. Another factor affecting plaque stability is intimal neovascularization. In the early phases of atherosclerosis, this is inhibited by AT_1_R blockers through their effects on toll-like receptors (TLRs), slowing down plaque development and improving plaque stability in experimental atherosclerosis [139]. AT_1_R (in rodents AT_1a_R) in atherosclerotic plaques mediates macrophage trapping, cytokine release, inflammation, oxidative stress, and matrix metalloproteinase activation, playing a crucial role in plaque vulnerability; deletion of AT_1a_R increased plaque stability [140].

In a surprising but promising work, Fraga-Silva et al. [31] have described that inflammatory processes could be inhibited in established atherosclerotic plaques when treated with the product of ACE2; the Ang (1–7) peptide activates the Mas receptor. Somewhat earlier, Dong et al. [141] published that Ang (1–7) improved plaque stability in an animal model of atherosclerosis. ACE2 expression and activity have been found in advanced human carotid atherosclerotic plaques, and it seems that it forms there a tissue-specific negative feedback regulator mechanism, counteracting the pro-atherosclerotic effect of local RAS [142]. Drugs slowing down plaque development might be developed based on these actions.

## 12. Vascular Wall Remodeling, Aortic Aneurysms

Aortas of mice kept on a high-fat diet undergo an outward wall remodeling that histologically resembles those found in obese patients and are generally thought to represent an initial form of microaneurysm development. The role of Ang II in this process is shown by the fact that the application of the angiotensin receptor blocker telmisartan attenuated this pathological transformation. This compound successfully prevented aortic dilatation and preserved elastin content. It was achieved by reducing the elevated expression of matrix metalloproteinases type 2 and 9, as well as TNFα [143].

The contribution of Ang II to this form of very serious atherosclerotic wall remodeling has also been demonstrated in Apo E^−/−^ mice fed a hypercholesterolemic diet. Chronic Ang II infusion induces aortic aneurysms in these animals, which can be prevented by the co-infusion of AT_1_R blockers. Some insight into the cellular pathomechanism is provided by the fact that co-infusion with the biased AT_1_R agonist TRV120027, which stimulates only the ß-arrestin-dependent mechanisms while inhibiting the cellular signaling pathways attached to G _q/11_ activation, prevented aneurysm development as effectively as the angiotensin receptor blockers themselves. Further analysis demonstrated that a higher level of protein production in the wall of animals with intact beta-arrestin function prevented the thinning of the wall and delayed fatal rupture [144]. In aneurysms induced by Ang II infusion in mice, an interplay between the angiotensin type 1 receptor and the lectin-like oxidized low-density lipoprotein receptor (LOX-1) is present. Knocking out the LOX-1 gene (Olr1) substantially elevated rupture events by reducing adventitial fibroblast proliferation and collagen synthesis [145]. Another investigation demonstrated that in hypercholesterolemic Ang II-infused mice, aneurysm development is the result of a direct effect of the agonist on the aortic wall, which develops independently of blood pressure elevation [146]. Antagonism of the AT_1_R-stimulated pro-atherosclerotic actions by the AT_2_Rs seems to be present even in aortic aneurysm formation [147].

Even aneurysms of different origin, e.g., induced in mice by a Marfan-like genetic trait, responded positively to angiotensin receptor blocker treatment [148]. In a recent study on fibrillin hypomorphic mice (*Fbn1^mgR^*^/*mgR*^), which develop a Marfan-like syndrome with dissecting aneurysm, the AT_1_R inhibitor losartan normalized the transcriptomic cell profile, increasing the number of normal smooth muscle cell populations while decreasing populations characteristic for the Marfan syndrome [149].

It seems that Ang II promotes aneurysm development by increasing the mechanical burden on the wall due to hypertension and inducing chronic inflammatory processes in the wall. At the same time, stimulation of protein expression, smooth muscle proliferation, and increased connective tissue production slow down aneurysmal dilation and delay fatal rupture.

**Figure 2:**
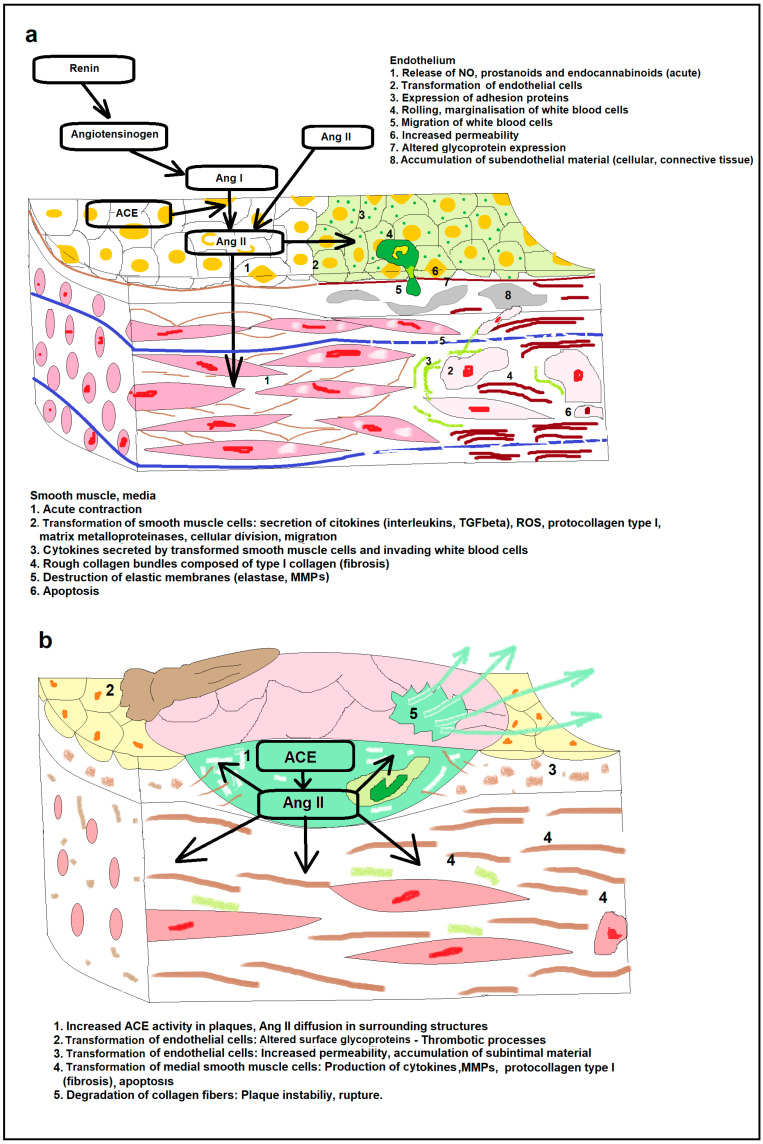
Ang II-induced pathologic remodeling of the vascular wall. (**a**). Remodeling of the endothelium and of the media (**b**). Role of Ang II in atherosclerotic vessel wall remodeling. Abbreviations: Ang I, angiotensin I; Ang II, Angiotensin II; ACE, angiotensin-converting enzyme; MMP, matrix metalloproteinase; TGFbeta, transforming growth factor beta; ROS, reactive oxygen species.

Processes of pathologic wall remodeling induced by Ang II are demonstrated in Figure 2.

## 13. Aging and Longevity

Deterioration of the vascular system is one of the most important alterations during the aging process of the human body. Chronic inflammation seems to be a key element of vascular wall aging [108]. Both in vitro and in vivo observations prove that the omnipresent inflammatory agonist of the vascular wall, Ang II, is an important factor that promotes the aging process [106,108]. Ang II stimulates cellular pathways leading toward apoptosis both in endothelial and vascular smooth muscle cells [150]. Interference of the AT_1_R signaling pathway with known pathways of cellular longevity has been proven in different vascular specimens. Disruption of the AT_1_R signaling pathway elevates the activity of the NAD+-dependent protein deacetylase SIRT3; it reduces reactive oxygen species (ROS) production and reduces damage to mitochondrial DNA, which is strongly connected to the cellular senescence process [151].

Increasing the rigidity of large elastic arteries close to the heart with deteriorating Windkessel function is an important factor in the reduction of maximum cardiac output during physical exercise in aged individuals. Loss of elasticity elevates ventricular and aortic pressures in the late ejection period and reduces the mechanical effectivity of heart contractions. Practically all cellular events of central artery rigidity in aged people are promoted by Ang II [152]. The decreasing endothelial dilation ability of aged vessels also seems to be connected, at least partially, to the local effect of Ang II [7].

The beneficial effects of resveratrol on vascular aging seem to be explained by its altering the balance between ACE and ACE2 expression in favor of producing the vasodilator Ang (1–7) [153].

Chronic administration of the ACE blocker captopril has been found to induce a small but significant elevation in the lifespan of female mice [154]. Recent publications seriously discuss the possibility of slowing down the vascular aging process by the wide application of RAS inhibitors [155]. The wide use of ACE blockers, however, is not without some risk. ACE is also an amyloid-degrading enzyme; the accumulation of amyloid in the brain causes serious neurodegeneration. Fortunately, several other amyloid-degrading enzymes do exist in the human tissues [156].

Finally, we can mention a far-reaching statement published by Kumar et al., raising wide interest and triggering a sharp debate [157]. According to them, Ang II may have a general biological effect throughout the animal kingdom, promoting the aging processes and reducing longevity.

**Figure 3:**
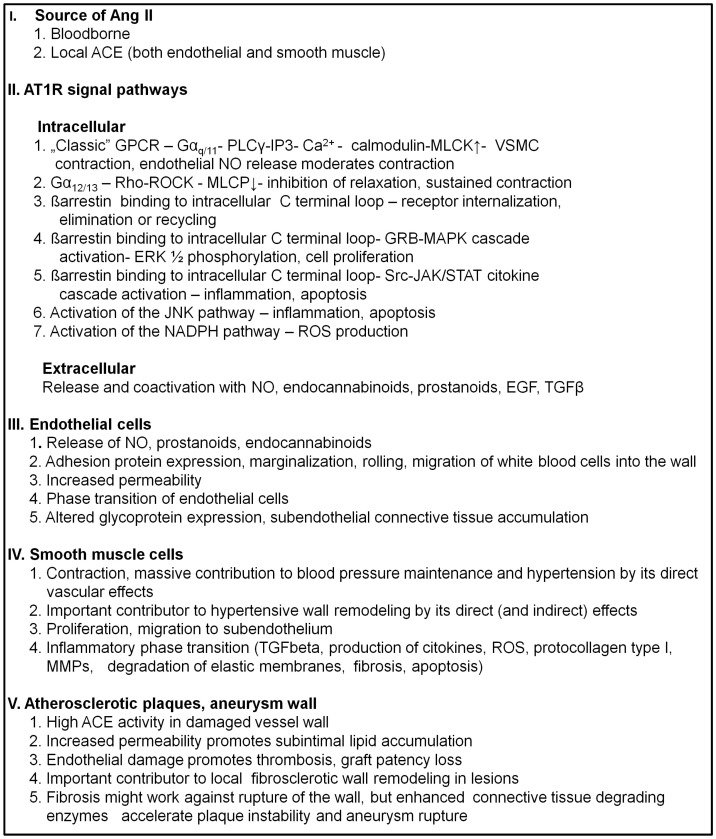
A schematic, concise description of direct angiotensin II effects on the vascular wall. Abbreviations: Ang II, angiotensin II; ACE, angiotensin-converting enzyme; AT_1_R, angiotensin type 1 receptor; GPCR, G protein-coupled receptor; G_α_, α subunit of heterotrimeric G protein; PLC, phospholipase C, IP3, inositol triphosphate; MLCK, myosin light chain kinase; VSMC, vascular smooth muscle cell; NO, nitric oxide; Rho, small GTPase; ROCK, Rho-associated kinase; MLCP, myosin light chain phosphatase; GRB, growth receptor binding protein; MAPK, mitogen-activated protein kinase; ERK1/2, extracellular signal-regulated kinases; Src, nonreceptor tyrosine kinase; JAK, Janus kinase; STAT, signal transducer and activator of transcription; JNK, c-Jun NH_2_-terminal kinase; NADPH, nicotinamide adenine dinucleotide phosphate, oxidoreductase coenzyme; ROS, reactive oxygen species; TGFß, transforming growth factor type β; EGF, epithelial growth factor; MMP, matrix metalloproteinase.

A schematic and concise description of direct angiotensin II effects on the vascular wall is listed in Figure 3.

## 14. Conclusions

We can conclude that Ang II has very diverse actions on the vascular wall. Both types of angiotensin receptors and ACE are expressed by the vessel wall’s two main cellular components, the endothelial cells and the vascular smooth muscle cells. A substantial part of this agonist will be locally synthesized. Its cellular actions are exerted through very complicated signal transduction mechanisms, intermingling with a large number of important cellular functions in different cell types. These effects have been optimized for situations very different from the challenges modern man has to face. Ang II’s functions are mainly beneficial. It helps keep vascular tone and blood pressure and controls the expression of important signal proteins and connective tissue components. In an emergency, e.g., in exsiccosis, blood loss, or blood pressure drop due to toxicosis, in situations which historically had been the most frequent causes of death, Ang II signaling serves as one of the most important mechanisms that helps keep body fluids and blood pressure at levels compatible with the continuation of life. Even fibrosis can be beneficial; it sacrifices hemodynamically optimal elasticity but strengthens the vascular wall when its mechanical integrity is endangered by mechanical factors or by tissue-diluting microbial attack.

Possibly for these reasons, a genetically determined Ang II production somewhat overrides the needs of common present-day requirements. Combined with other over-regulated blood pressure control mechanisms, this now forms an important pathologic component of several serious cardiovascular and kidney diseases.

We can distinguish between the immediate and lasting direct vascular effects of Ang II. The direct effect is exerted mostly through the AT_1_R, which induces powerful vascular smooth muscle contraction moderated somewhat by endothelial- and endocannabinoid-mediated dilation. An immediate Ang (1–7) induced dilation with the contribution of AT_2_R can also be present. The former action is conveyed through the classic G protein-coupled receptor mechanism through the G_q/11_/PLC/IP_3_/Ca^2+^/calmodulin/MLCK reaction pathway. At the same time, an additional G_12/13_/RhoA/ROCK/MLCP inhibition pathway ensures sustained contraction. Increased intracellular Ca^2+^ activates PKCs, for which phosphorylation sites are present on many proteins, massively influencing many cellular functions. A little bit later, ß-arrestin association with the intracellular C-terminal segment of the receptor molecule initiates endocytosis as well as lasting nuclear effects with activation of the MAPK/ERK 1/2 and Src/JAK/STAT pathways, changing the expression of a large number of proteins. Activation of the JNK pathway has also been described. Substantial expression changes, even cellular phase transition, can be the result.

Higher than optimal stimulation of the AT_1_Rs induces chronic inflammation with elevated NF-κB activities and the phase transition of endothelial and smooth muscle cells. This is combined with the expression of adhesion proteins, chemoattractants, other cytokines, enzymes for degradation of existing elastin and collagen structures, and protocollagen to form new, rough collagen bundles. The degradation of elastic tissue by matrix metalloproteinases increases the rigidity of the wall. ROS-producing processes will be stimulated. Cells damaged by chronic inflammation will apoptotize, and pathologic processes of hypertensive wall remodeling, arteriosclerotic plaque development and rupture, and aneurysm formation will be accelerated.

Deteriorating endothelial dilation increases hemodynamic resistance. Vascular rigidity increases the pulsatile stress on the arterial wall, and high intraluminal pressure (an indirect effect of Ang II), together with the local Ang II effect, will promote the thickening of the wall, further reducing the lumen of small arteries. If no intact smooth muscle and elastica layers are at hand any longer to prevent slow morphological dilation of the aorta, increased pressure in the late ejection phase makes the development of heart failure almost unavoidable.

The angiotensin receptors have been subjected to intensive pharmacological studies. Important developments in the last years are the demonstration of the possibility of constructing drugs with “biased” effects, with separate actions on the G protein or on the arrestin-dependent signal pathways. Selective agonists of the AT_2_R are under development. Even hopes are raised for pharmacological interventions into the complicated intracellular signal pathways. Promising are studies demonstrating the effect of these inhibitors on longevity.

## Data Availability

Not applicable.
